# Natural course of AIDS following HIV-2 infection

**DOI:** 10.1007/s15010-023-02143-3

**Published:** 2023-12-12

**Authors:** Hannah Fuhr, Maximilian Christopeit

**Affiliations:** 1 Outpatient Practice for Primary Care and Infectious Disease (Gemeinschaftspraxis, Drs Frietsch, Roll, Marquardt), Stuttgart, Germany; 2 Medical Clinic, Department of Hematology, Oncology, Clinical Immunology and Rheumatology, University Hospital Tübingen, Tübingen, Germany; 3 Department of Medicine II (Oncology, Hematology and Bone Marrow Transplantation With Section Pneumology), University Cancer Center Hamburg, University Medical Center Hamburg-Eppendorf, Martinistraße 52, 20246 Hamburg, Germany

**Keywords:** HIV2, AIDS, Lymphoma

## Abstract

In a 21-year-old female, AIDS following infection with HIV-2 was diagnosed alongside an HIV-associated high-grade B cell lymphoma. Treatment of HIV-2 with dolutegravir, emtricitabine, and tenofovir resulted in viral suppression and slow recovery of CD4 cell counts. Treatment of lymphoma caused significant adverse effects but led to complete remission. The patient denied sexual activity and intravenous drug abuse. The patient had been born to an HIV-2-positive mother but appropriate perinatal testing based on national guidelines had remained negative. This case recapitulates the natural course of HIV-2 infection.

## Case report

A 21-year-old female presented with a 3-week history of lower back pain that had exacerbated 1 week before presentation. The pain radiated into both lower extremities. On review of systems, she endorsed nausea and vomiting, loss of appetite, a history of unintentional weight loss of 10 kg during the last year, and night sweats but no fever. Bone marrow analysis revealed an HIV-associated high-grade B cell lymphoma, Ann Arbor stage IVB with intermediate high international prognostic index and high age-adjusted international prognostic index, molecularly resembling Burkitt lymphoma. Detection of HIV-2 DNA, CMV viremia, pulmonary Aspergillosis with Asp. niger and Pneumocystis jiroveci pneumonia completed the picture of AIDS. At the time of initial HIV diagnosis the HIV viral load was 6300 cop/mL and the CD4 count was 43/μL.

Lymphoma treatment with polychemotherapy [1] was tolerated poorly, necessitating frequent delays and dose reductions. It resulted in complete remission. Despite reported good adherence to ART (dolutegravir, emtricitabine, tenofovir), immune reconstitution was slow, as tends to be the case for HIV-2 [2], and low levels of viremia persisted. Resistance testing showed susceptibility to all PI, all NRTI, all INSTI and resistance against all NNRTI as well as fosamprenavir and tipranavir. Atazanavir was not recommended. Accompanying strategies to handle the before-mentioned opportunistic infections were uneventful, as were prophylactic measures.

Both route and timing of infection remain obscure. The patient assured different physicians of a negative sexual history and denied intravenous drug use. Even in the case of sexual activity, infection with HIV-1 would have been more likely than infection with HIV-2 in the areas where she had lived. Interestingly, this patient had been born via cesarean section to an HIV-2-positive mother. We were unable to ascertain the mother’s viral load during delivery. It remains obscure whether postnatal HIV transmission prophylaxis was administered. Our patient was born and raised in Germany and visited her mother’s native West Africa (Ghana) only once for a few weeks at the age of three. Repeated attempts to detect HIV-2 were unsuccessful during her early years of life. German–Austrian guidelines consider perinatal transmission ruled out if an exposed infant has two negative HIV PCR tests after one and 3 months of life. Our patient showed appropriately waning maternal HIV-2 antibodies (> 1:4096 at birth, 1:256 at 3 months of age, and 1:16 at 6 months of age). In addition, HIV-2 DNA PCR results, performed at a National Reference Center of a university hospital, were negative at 3 and 6 months of age. Both antibodies and PCR were negative at 12 and 18 months of age, with a 4th generation HIV-1/2 AK + p24 Ag test (HIV Integral DADE BEHRING). Of note, the patient did describe a history of chronic sinusitis requiring surgeries as well as an episode of varicella zoster around age 12.

This case highlights the natural course of AIDS following HIV-2 infection in that it may take years and perhaps even decades to fully develop. Testing of individuals at risk, particularly in the presence of HIV indicator conditions such as varicella zoster, must be considered, even if initial tests remain negative. Since levels of viremia of HIV-2 are frequently low, PCR assays might need to be supplemented by appropriate antibody tests for accurate and timely diagnosis of HIV-2 infection.

## Data Availability

Not applicable.
